# Chitosan nanoparticles having higher degree of acetylation induce resistance against pearl millet downy mildew through nitric oxide generation

**DOI:** 10.1038/s41598-017-19016-z

**Published:** 2018-02-06

**Authors:** Chandra Nayaka Siddaiah, Keelara Veerappa Harish Prasanth, Niranjan Raj Satyanarayana, Venkataramana Mudili, Vijai Kumar Gupta, Naveen Kumar Kalagatur, Tara Satyavati, Xiao-Feng Dai, Jie-Yin Chen, Andrei Mocan, Bhim Pratap Singh, Rakesh Kumar Srivastava

**Affiliations:** 1 Department of Studies in Biotechnology, University of Mysore, Manasagangotri, Mysore, 570006 Karnataka India; 2 Department of Biochemistry, Central Food Technological Research Institute, Council of Scientific and Industrial Research, Mysore, 570 020 Karnataka India; 3 Department of Studies in Microbiology, Karnataka State Open University, Mukthagangotri, Mysore, 570006 Karnataka India; 4 Microbiology Division, DRDO-BU-Centre for Life sciences, Bharathiar University Campus, Coimbatore, 641046 Tamil Nadu India; 5 Department of Chemistry and Biotechnology, School of Science, Tallinn University of Technology, Tallinn, Estonia; 6 All India Coordinated Research Project on Pearl Millet, Indian Council of Agricultural Research, Mandor, Jodhpur, 342 304 Rajasthan India; 7 Institute of Food Science and Technology, Chinese Academy of Agricultural Sciences, Beijing, 100193 China; 8 Department of Pharmaceutical Botany, Faculty of Pharmacy, University of Medicine and Pharmacy “Iuliu Hațieganu”, Ghe. Marinescu 23, 400337 Cluj-Napoca, Romania; 9 Molecular Microbiology and Systematics Laboratory, Department of Biotechnology, Mizoram University, Mizoram, India; 10 International Crops Research Institute for the Semi-Arid Tropics (ICRISAT), Patancheru, 502324 Telangana India

## Abstract

Downy mildew of pearl millet caused by the biotrophic oomycete *Sclerospora graminicola* is the most devastating disease which impairs pearl millet production causing huge yield and monetary losses. Chitosan nanoparticles (CNP) were synthesized from low molecular weight chitosan having higher degree of acetylation was evaluated for their efficacy against downy mildew disease of pearl millet caused by *Sclerospora graminicola*. Laboratory studies showed that CNP seed treatment significantly enhanced pearl millet seed germination percentage and seedling vigor compared to the control. Seed treatment with CNP induced systemic and durable resistance and showed significant downy mildew protection under greenhouse conditions in comparison to the untreated control. Seed treatment with CNP showed changes in gene expression profiles wherein expression of genes of phenylalanine ammonia lyase, peroxidase, polyphenoloxidase, catalase and superoxide dismutase were highly upregulated. CNP treatment resulted in earlier and higher expression of the pathogenesis related proteins PR1 and PR5. Downy mildew protective effect offered by CNP was found to be modulated by nitric oxide and treatment with CNP along with NO inhibitors cPTIO completely abolished the gene expression of defense enzymes and PR proteins. Further, comparative analysis of CNP with Chitosan revealed that the very small dosage of CNP performed at par with recommended dose of Chitosan for downy mildew management.

## Introduction

Pearl millet is a drought tolerant, coarse cereal grown on infertile soils under water-limited conditions serving as a staple food for millions of poor people of semi-arid tropical regions of Africa and Asia. India has 10 million ha under pearl millet cultivation and the grain is the major source of human diet and the stover forms the basis of livestock ration^1^. Nutritional aspects of pearl millet are unique when compared to other cereal crops. Feeding trials have shown that millet is nutritionally superior to maize and rice. Among the diseases of pearl millet, downy mildew caused by the biotrophic oomycete Sclerospora graminicola is the most devastating disease responsible for wide-spread yield and economic losses^2^. *Sclerospora graminicola* is highly variable and has the ability to quickly adapt to the newly released hybrids and consequently breaks down the host resistance thereby leading to regular withdrawal of the downy mildew-resistant hybrids. Chemical treatment is not feasible for a poor crop like pearl millet and the currently used chemicals are known to cause harmful effects on both human and environmental health. Consequently, exploring more safe and eco-friendly options like stimulating the innate immunity or inducing resistance have become potential alternatives for management of pearl millet downy mildew.

The derivative of chitin, Chitosan, is a β-1,4-linked glucosamine which has been shown to have resistance stimulating ability against several plant pathogens. Specifically, chitosan pre-treatment primes the host plant defense responses by inducing earlier and higher phytoalexin synthesis, lignification, callose deposition, activities of reactive oxygen species, enhanced defense enzymes and PR proteins^3,4^. Plant protective efficiency of chitosan against a wide range of phytopathogens has been well established in several crops^4,5^.

Conversion of chitosan to nanochitosan presents many characteristics like biocompatibility, biodegradability and reduced toxicity which are best suited for effective delivery of the elicitor. In comparison to chitosan, nanochitosan has altered physiochemical properties like size, surface area, cationic nature which consequently alters the biological activity^6^. Ability of nanochitosan in eliciting resistance against various plant diseases has been demonstrated in many host-pathogen interactions. Cu-chitosan nanoparticles effectively controlled tomato early blight and *Fusarium* wilt^7^. Chitosan nanoparticles effectively suppressed of rice and finger millet blast fungus *Pyricularia grisea*^8,9^.

The aim of the present study was to comparatively evaluate chitosan and chitosan nanoparticles (CNP) synthesized specially from water soluble patent granted low molecular weight chitosan having higher degree of acetylation, for their defense stimulating efficiency against pearl millet downy mildew disease and to understand some of the biochemical and molecular events underlying the induced resistance process.

## Results

### Preparation of chitosan nanoparticles

**Figure 1:**
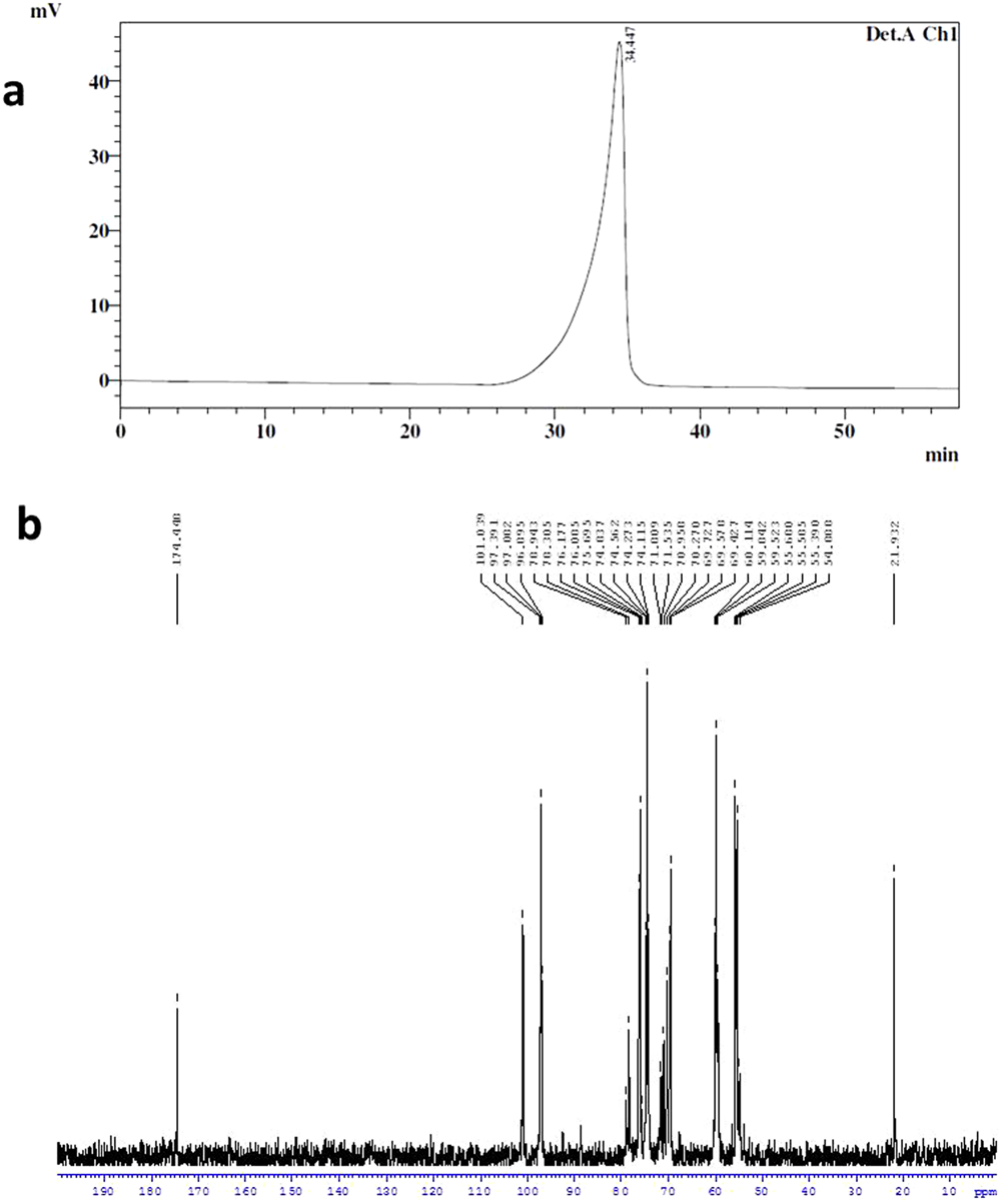
Low molecular weight (LMWC) used for nanoparticles preparation (**a**) HPLC-SEC profile showing single peak having MW ~20 kDa and (**b**) 13C-NMR spectrum for LMWC having ~70% acetylation. 13C-NMR (δ, ppm) C1: 101.03, 97.08; C2: 55.68; C3: 69.72 (69–71); C4: 78.94, 78.30, 76.05; C5: 74.2; C6: 59.84; C=O: 174.44; CH_3_: 21.93.

To obtain viscosity free and readily water soluble low molecular weight, LMWC^10^ with higher degree of acetylation (Even though, native chitosan used to prepare LMWC was having ~20% acetylation, LMWC thus prepared showed ~70% acetylation, Fig. 1) was adopted to prepare nanoparticles for this study. Further, the nanoparticles were well characterized and reported elsewhere previously^11^.

Effect of CNP on seed germination and seedling vigor of pearl millet under laboratory conditions.

The effect of CNP on pearl millet seed germination and seedling vigor was assessed by preparing different concentrations of CNP and compared with untreated control and chitosan. Seed treatment with 250 mg kg^−1^ concentration of CNP offered the best results in terms of seed growth parameters. 250 mg kg^−1^ concentration of CNP resulted in 97% pearl millet seed germination and 1937 seedling vigor which were significantly higher than the chitosan treatment and the untreated control. Chitosan treatment recorded 89% seed germination and 1917 seedling vigor. The untreated control showed 82% germination and 1866 seedling vigor (Table 2).

### Efficiency of CNP to elicit resistance against downy mildew under greenhouse

**Figure 2:**
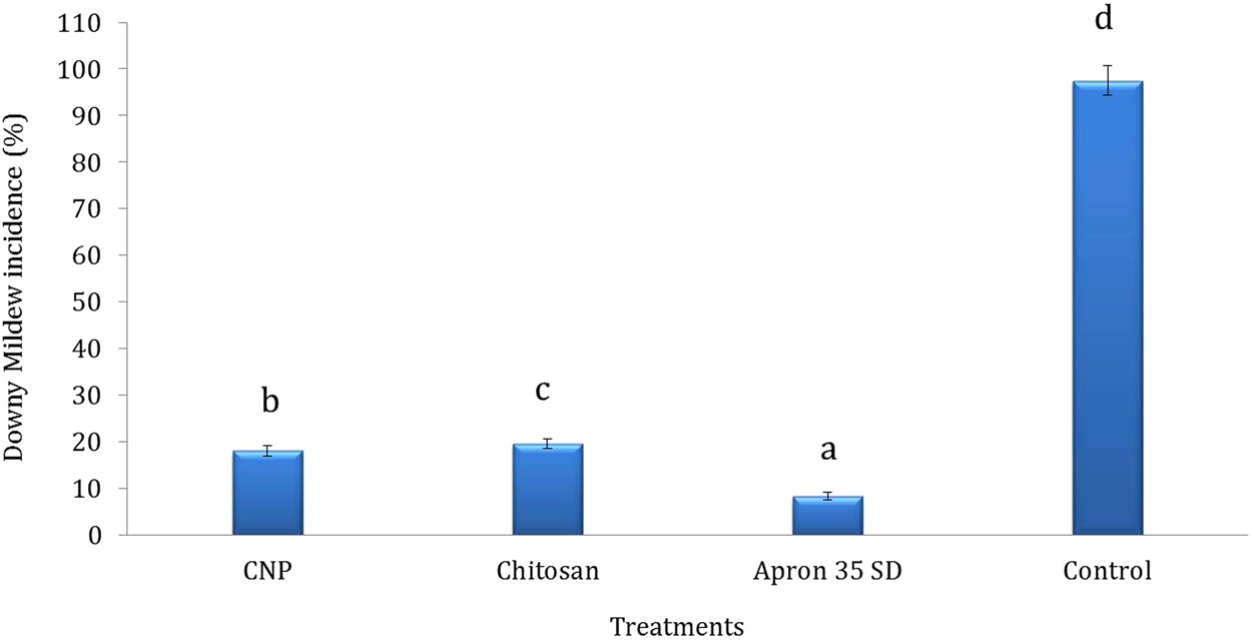
Effect of seed treatment with CNP on downy mildew disease incidence of pearl millet under greenhouse conditons. Pearl millet seeds of 7042 S (highly downy mildew susceptible variety) were treated with different concentration of CNP and were sown in earthen pots and the emerging seedlings were inoculated with *Sclerospora graminicola* zoospores at 4 × 10^4^/ml concentration. After 30 days of sowing downy mildew incidence was recorded. Greenhouse experiments were carried out in four replicates and repeated twice. Bars indicate the standard error; means with different superscripts are significantly different as indicated by Tukey’s HSD (P = 0.05).

Since 250 mg kg (−1) concentration of CNP was found to be the optimum connotation for seed treatment it was further tested for downy mildew disease reaction under greenhouse conditions, along with chitosan, apron and untreated control as checks. CNP recorded 18.1% downy mildew disease incidence which was lowest among the tested treatments. Chitosan treatment showed 19.6% downy mildew incidence. Apron and untreated control showed 8.3 and 97.5% downy mildew disease incidence respectively (Fig. 2).

### Demonstration of the nature of resistance induction by CNP

**Figure 3:**
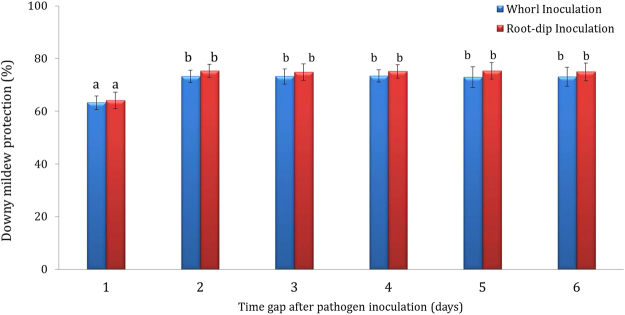
Demonstrations of systemic nature of resistance induction by CNP by spatiotemporal separation of the inducer and pathogen inoculation. Emerging pearl millet seedlings raised from 7042 S seeds treated with CNP were challenge-inoculated with the zoospore suspension of *Sclerospora graminicola* by adding 4–5 drops (0.5 ml) to the leaf whorl of each plant at intervals of 1, 2, 3, 4, 5 and 6 days between the seedling emergence and pathogen inoculation in different sets of plants. After 30 days of sowing downy mildew incidence was recorded. The experiments were carried out in four replicates and repeated twice. Bars indicate the standard error; means with different superscripts are significantly different as indicated by Tukey’s HSD (P = 0.05).

CNP treatment was further tested to the nature of protection offered under greenhouse conditions. The nature of resistance elicitation was demonstrated following the spatial and temporal separation method. The results showed that the resistance elicited by CNP treatment to pearl millet seeds is systemic. Initially, when the time gap between seed treatment and pathogen inoculation was 1 day, CNP treatment resulted in 63% downy mildew protection. The protection percentage was raised to 73% on the second day which consistently maintained throughout the experimental period thus indicating that a minimum 2 days were required for the total resistance build up. The trend was similar in the second set of experiments where the inducer treatment was given as root dip inoculation. Initially, at 1-day gap, the protection offered was 64%. This shot up to 75% on the second-day gap. This protection percentage was sustained throughout the experimental period (Fig. 3).

## Biochemical studies

### Enzyme assays

#### Phenylalanine ammonia-lyase assay

**Figure 4:**
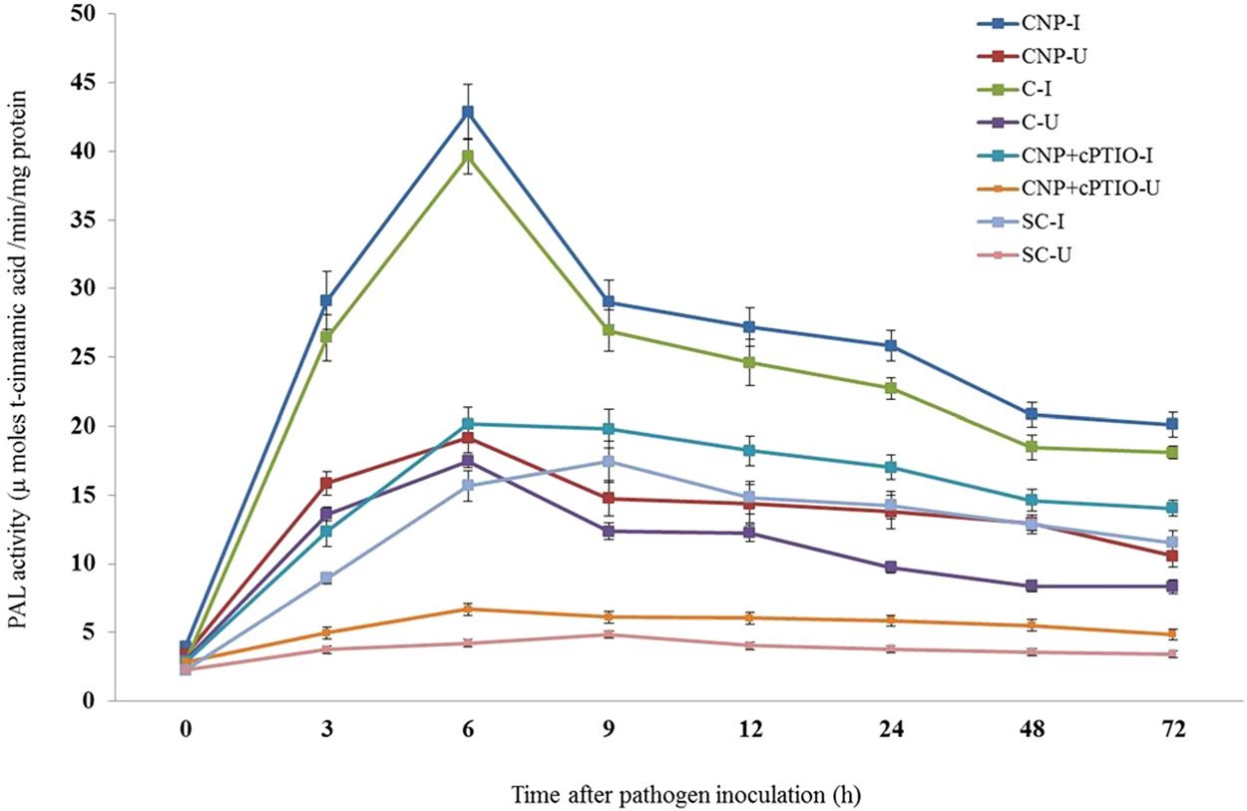
Pattern of accumulation of Phenylalanine ammonia lyase in two-day old pearl millet seedlings with (inoculated) or without (uninoculated) *Sclerospora graminicola* inoculation. Chitosan-Seedlings treated with Chitosan, CNP-Seedlings treated with the chitosan nanoparticles, CNP + cPTIO – seedlings treated with CNP followed by cPTIO treatment, Control: Seedlings of downy mildew susceptible cultivar. Phenylalanine ammonia lyase was determined as activity was determined as the amount of t-cinnamic acid formed from L-Phenylalanine per mg of protein per min measured spectrophotometrically at a wavelength of 290 nm. Data of enzyme activity are means ± SE of three different experiments. The values were the means of three replicates of three different experiments. Bars indicate the standard error as indicated by Tukey’s HSD (P = 0.05).

Constitutive PAL activity was observed in all categories of seedlings with or without pathogen inoculation. At all tested time points, PAL activity was significantly higher in pathogen-inoculated seedlings compared to the uninoculated seedlings. In all the tested seedlings PAL activity peaked at 6 hpi as against the control seedlings in which PAL activity peaked at 9 hpi. At 6 hpi, among the treated seedlings maximum PAL activity was recorded by CNP treated seedlings which showed 1.08, 2.12 and 2.73 folds higher than Chitosan treated, CNP + cPTIO and untreated control seedlings respectively. Further, PAL activity in inoculated CNP treated seedlings was 2.24 folds more than the uninoculated seedlings (Fig. 4).

#### Peroxidase assay

**Figure 5:**
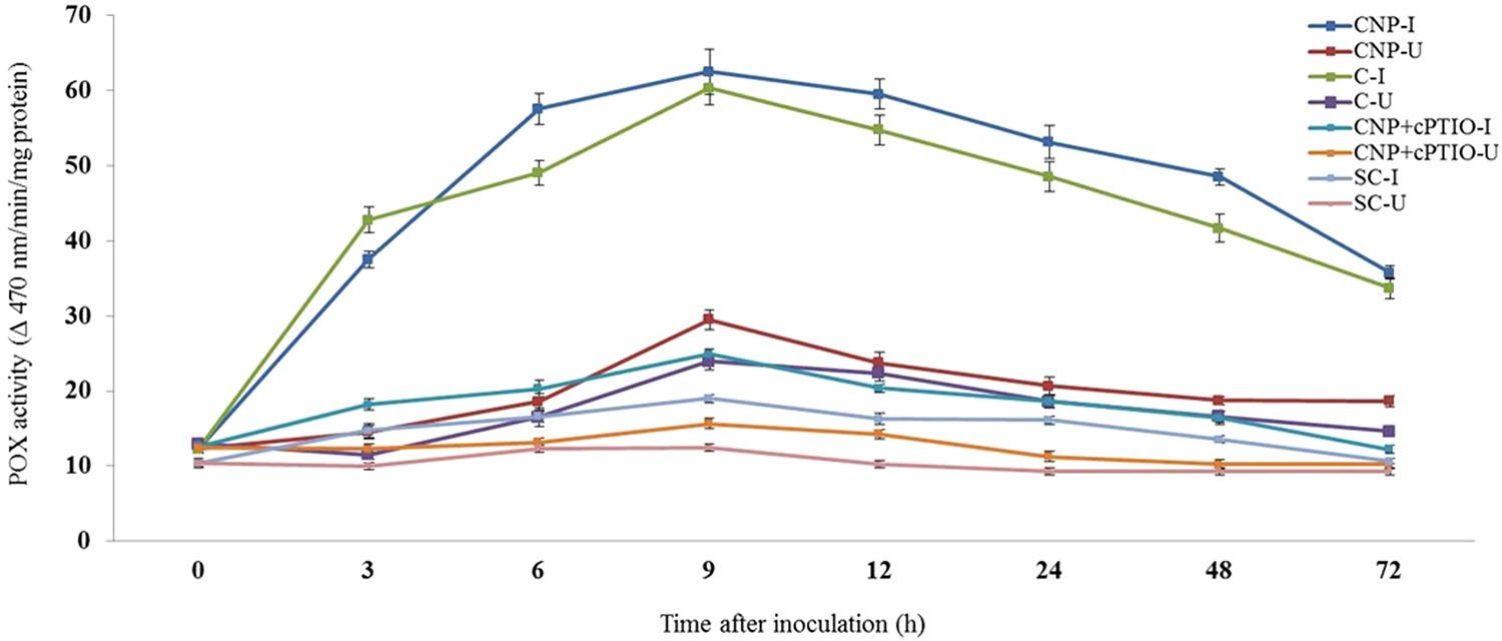
Pattern of accumulation of Peroxidase in two-day old pearl millet seedlings with (inoculated) or without (uninoculated) *Sclerospora graminicola* inoculation. Chitosan-Seedlings treated with Chitosan, CNP-Seedlings treated with the chitosan nanoparticles, CNP + cPTIO – seedlings treated with CNP followed by cPTIO treatment, Control: Seedlings of downy mildew susceptible cultivar. Peroxidase activity determined as the increase in absorbance recorded 470 nm. POX activity is expressed in terms of the change in A470 min^−1^ mg^−1^ protein. Data of enzyme activity are means ± SE of three different experiments. The values were the means of three replicates of three different experiments. Bars indicate the standard error as indicated by Tukey’s HSD (P = 0.05).

Constitutive POX activity was observed in all categories of seedlings with or without pathogen inoculation. At all tested time points, POX activity was significantly higher in pathogen-inoculated seedlings compared to the uninoculated seedlings. In all the tested seedlings POX activity gradually increased from 3 h peaked at 9 with or without pathogen inoculation. At 9 hpi, among the treated seedlings maximum POX activity was recorded by CNP treated seedlings which showed 1.03, 2.51 and 3.29 folds higher than Chitosan treated, CNP + cPTIO and untreated control seedlings respectively. Further, POX activity in inoculated CNP treated seedlings was 2.12 folds more than the uninoculated seedlings (Fig. 5).

#### Polyphenol oxidase assay

**Figure 6:**
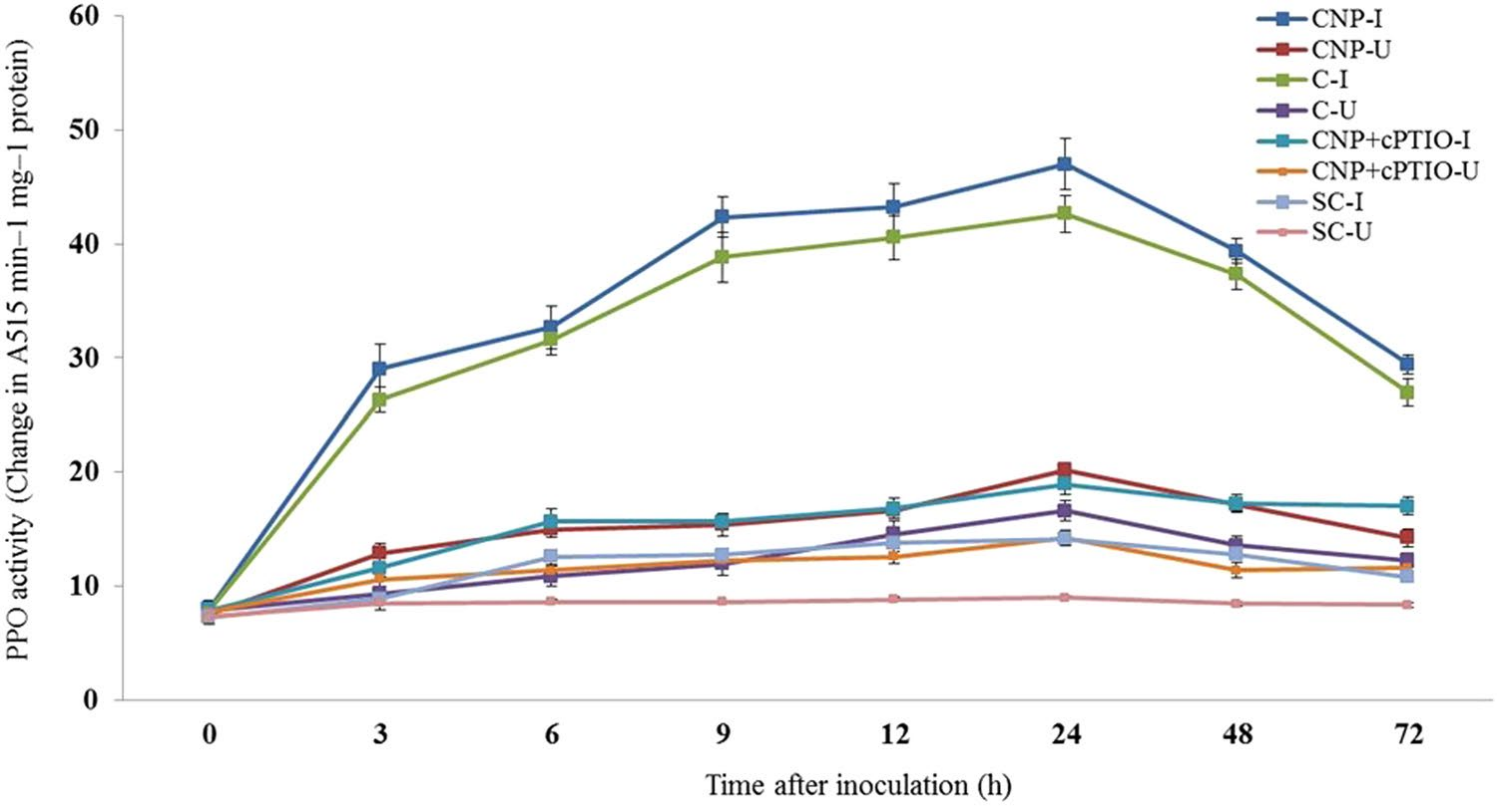
Pattern of accumulation of Polyphenol oxidase in two-day old pearl millet seedlings with (inoculated) or without (uninoculated) *Sclerospora graminicola* inoculation. Chitosan-Seedlings treated with Chitosan, CNP-Seedlings treated with the chitosan nanoparticles, CNP + cPTIO – seedlings treated with CNP followed by cPTIO treatment, Control: Seedlings of downy mildew susceptible cultivar. Polyphenol oxidase determined as increase in absorbance at 420 nm was recorded for 1 min. The results are expressed as the change in A per min per mg protein Data of enzyme activity are means ± SE of three different experiments. The values were the means of three replicates of three different experiments. Bars indicate the standard error as indicated by Tukey’s HSD (P = 0.05).

Constitutive PPO activity was observed in all categories of seedlings with or without pathogen inoculation. At all tested time points, PPO activity was significantly higher in pathogen-inoculated seedlings compared to the uninoculated seedlings. In all the tested seedlings PPO activity gradually increased from 3 h peaked at 24 with or without pathogen inoculation. At 24 hpi, among the treated seedlings maximum PPO activity was recorded by CNP treated seedlings which showed 1.10, 2.48 and 3.33 folds higher than Chitosan treated, CNP + cPTIO and untreated control seedlings respectively. Further, PPO activity in inoculated CNP treated seedlings was 2.33 folds more than the uninoculated seedlings (Fig. 6).

#### Superoxide dismutase assay

**Figure 7:**
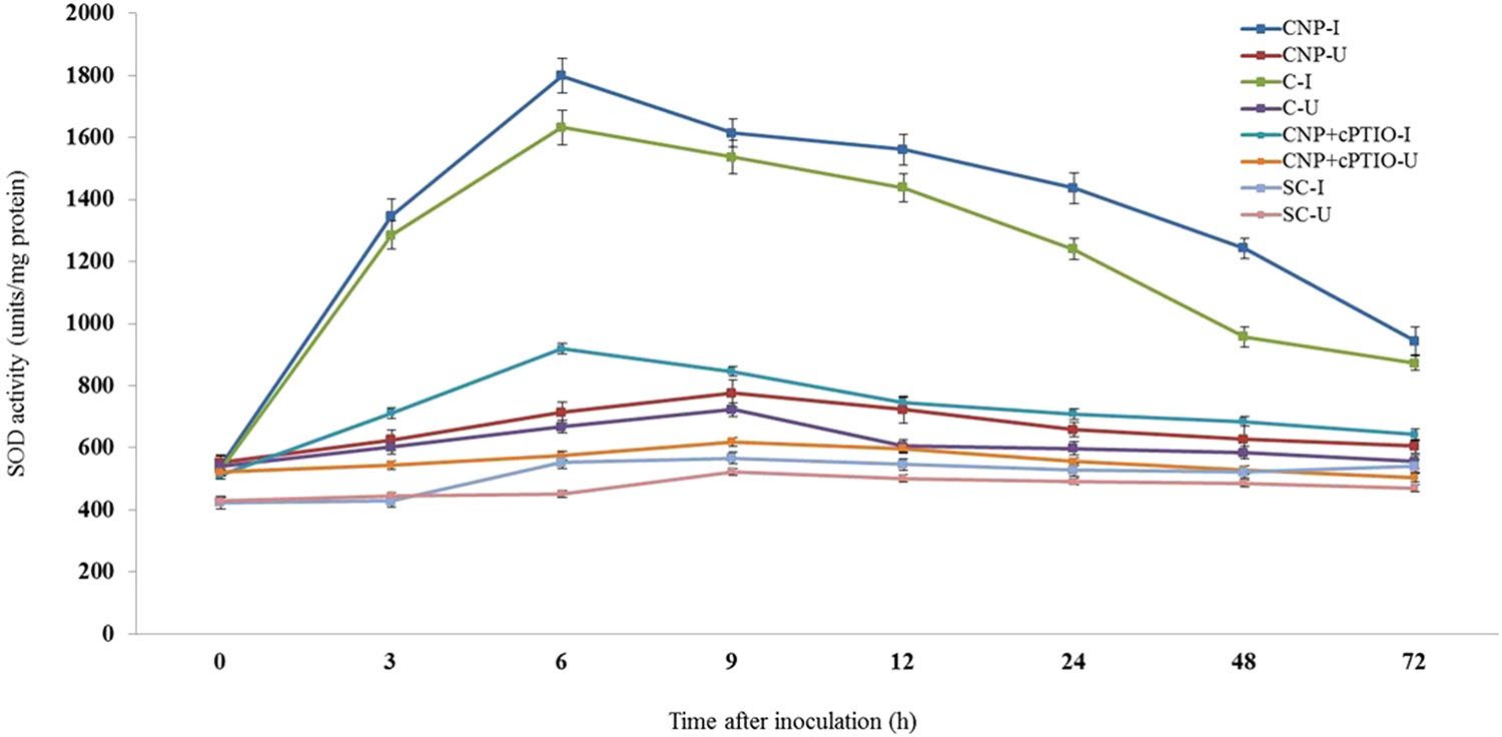
Pattern of accumulation of Superoxide dismutase in two-day old pearl millet seedlings with (inoculated) or without (uninoculated) *Sclerospora graminicola* inoculation. Chitosan-Seedlings treated with Chitosan, CNP-Seedlings treated with the chitosan nanoparticles, CNP + cPTIO – seedlings treated with CNP followed by cPTIO treatment, Control: Seedlings of downy mildew susceptible cultivar. Superoxide dismutase was determined as change in absorbance at 560 nm was recorded for 1 min. Data of enzyme activity are means ± SE of three different experiments. The values were the means of three replicates of three different experiments. Bars indicate the standard error as indicated by Tukey’s HSD (P = 0.05).

Constitutive SOD activity was observed in all categories of seedlings with or without pathogen inoculation. At all tested time points, SOD activity was significantly higher in pathogen-inoculated seedlings compared to the uninoculated seedlings. In all the tested seedlings SOD activity peaked at 6 hpi as against the control seedlings in which SOD activity peaked at 9 hpi. At 6 hpi, among the treated seedlings maximum SOD activity was recorded by CNP treated seedlings which showed 1.10, 1.96 and 3.09 folds higher than Chitosan treated, CNP + cPTIO and untreated control seedlings respectively. Further, SOD activity in inoculated CNP treated seedlings was 2.32 folds more than the uninoculated seedlings (Fig. 7).

#### Catalase assay

**Figure 8:**
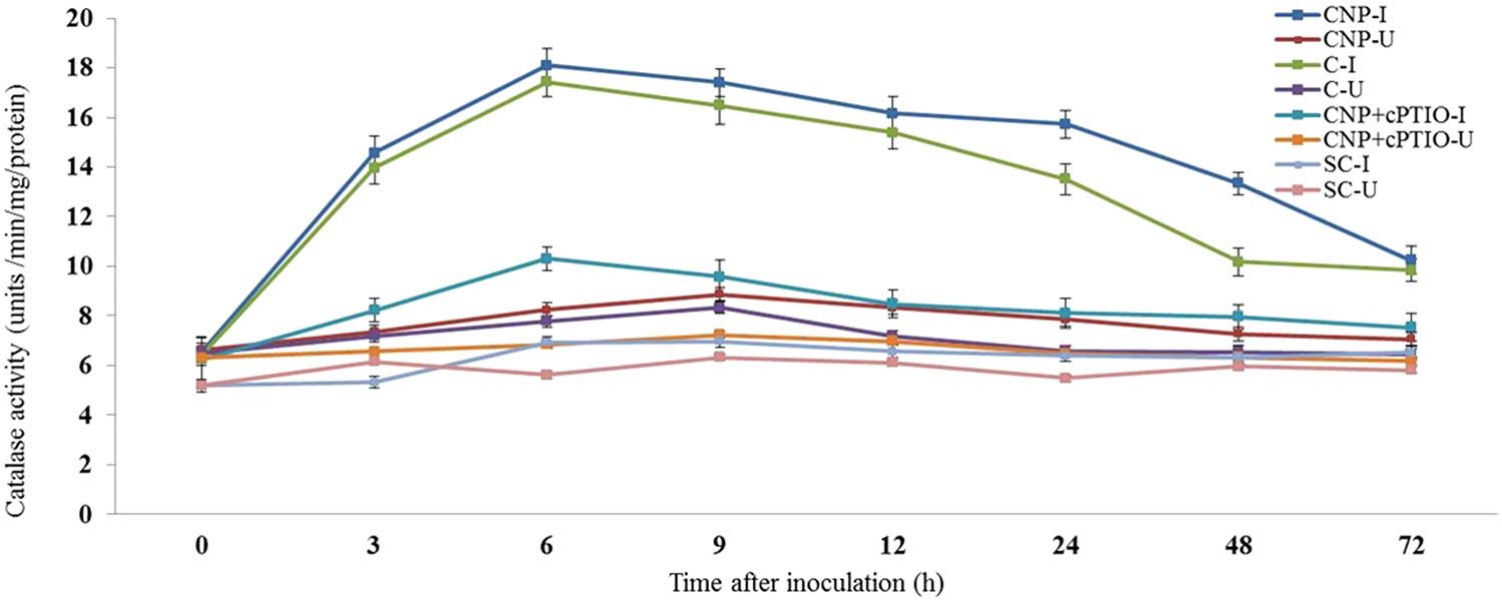
Pattern of accumulation of Catalase in two-day old pearl millet seedlings with (inoculated) or without (uninoculated) *Sclerospora graminicola* inoculation. Chitosan-Seedlings treated with Chitosan, CNP-Seedlings treated with the chitosan nanoparticles, CNP + cPTIO – seedlings treated with CNP followed by cPTIO treatment, Control: Seedlings of downy mildew susceptible cultivar. Catalase was determined as change in absorbance at 240 nm was recorded for 1 min. Data of enzyme activity are means ± SE of three different experiments. The values were the means of three replicates of three different experiments. Bars indicate the standard error as indicated by Tukey’s HSD (P = 0.05).

Constitutive CAT activity was observed in all categories of seedlings with or without pathogen inoculation. At all tested time points, CAT activity was significantly higher in pathogen-inoculated seedlings compared to the uninoculated seedlings. In all the tested seedlings CAT activity peaked at 6 hpi as against the control seedlings in which CAT activity peaked at 9 hpi. At 6 hpi, among the treated seedlings maximum CAT activity was recorded by CNP treated seedlings which showed 1.03, 1.76 and 2.59 folds higher than Chitosan treated, CNP + cPTIO and untreated control seedlings respectively. Further, CAT activity in inoculated CNP treated seedlings was 2.04 folds more than the uninoculated seedlings (Fig. 8).

#### Gene expression studies

Quantitative real-time PCR analysis (qPCR) for defense enzymes, and pathogenesis-related proteins.

Real-time PCR analysis was carried out to investigate the effect of different treatments on mRNA expression of defense-related genes in comparison untreated control with or without pathogen inoculation. The CNP treated pearl millet seedlings, with or without pathogen inoculation, showed rapid and significantly enhanced expression of PAL, POX, PPO, SOD, CAT, PR1, and PR5 genes in comparison with the untreated control seedlings. Constitutive levels of gene expression were observed for all the tested genes in all categories of seedlings, which gradually increased after pathogen inoculation.

**Figure 9:**
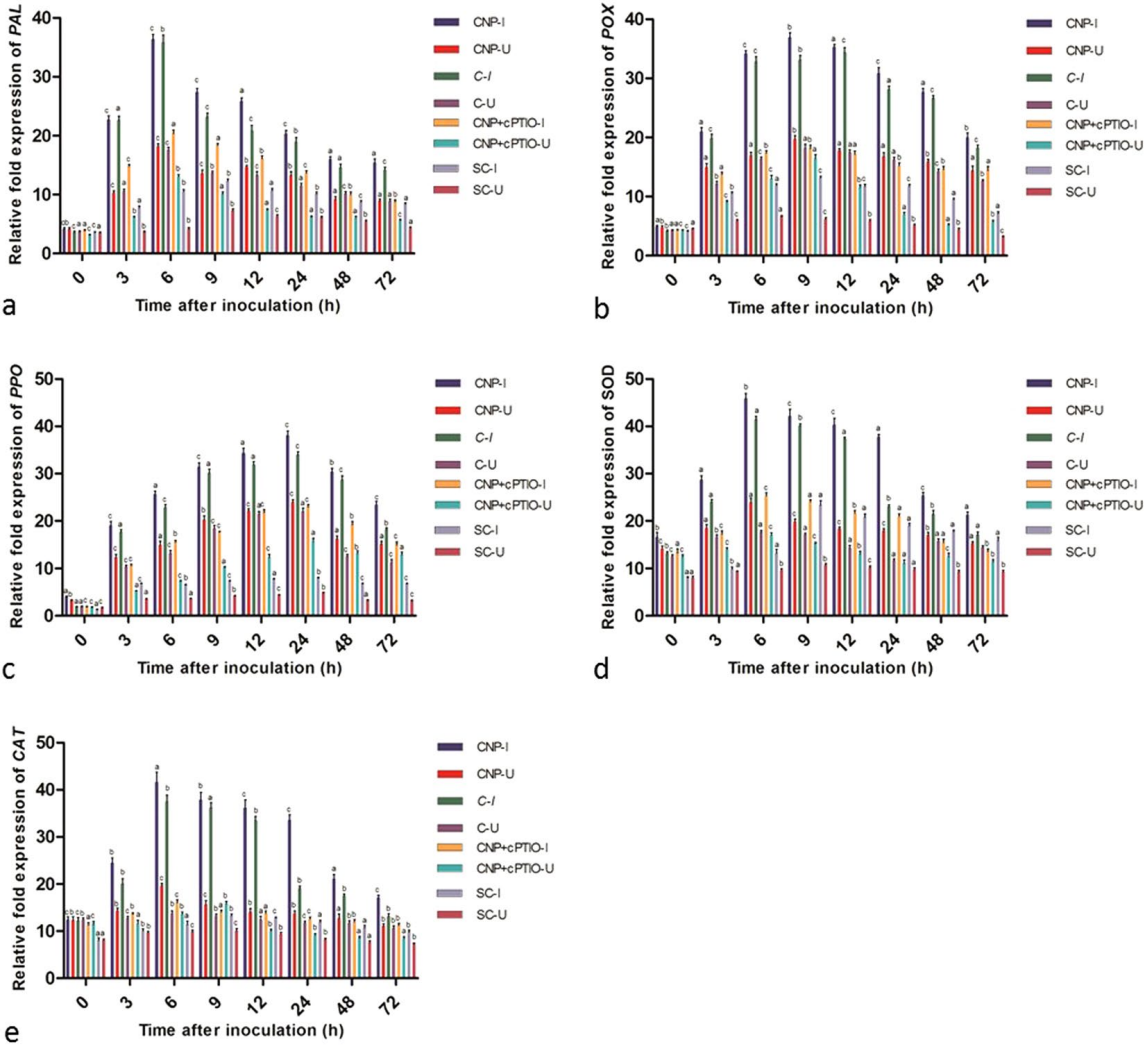
qRT-PCR determined relative expression of genes of various defense enzymes in two-day-old pearl millet seedlings with (I) or without (U) *Sclerospora graminicola* inoculation harvested 0, 3, 6, 9, 12, 24, 48, and 72 h. Chitosan-Seedlings treated with Chitosan, CNP-Seedlings treated with the chitosan nanoparticles, CNP + cPTIO – seedlings treated with CNP followed by cPTIO treatment, Control: Seedlings of downy mildew susceptible cultivar. (**a**) Phenylalanine ammonia lyase (**b**) Peroxidase (**c**) Polyphenol oxidase (**d**) Superoxide dismutase and (**e**) Catalase. Expression levels were measured by qPCR and normalized to the constitutive PP2A gene. Values are means of a single experiment carried out in triplicate. The bars indicate ± SE and the data were analyzed by one-way ANOVA followed by Tukey’s test and p-value *< or =0.05 was significant compared with control and ** < 0.01 significant with treated control.

After inoculation with the downy mildew pathogen PAL expression gradually increased and peaked at 6 hpi in treated seedlings, whereas in control seedlings PAL activity peaked at 9 hpi. At 6 hpi, among the treatments, maximum PAL expression was recorded in CNP treated seedlings which was 1.01, 1.78, and 3.45 folds higher than the Chitosan treated, CNP + cPTIO treated and the untreated control respectively (Fig. 9a).

POX gene expression gradually increased from 3 h onwards and maximum POX transcript level was recorded at 9 hai which decreased thereafter. At 9 hpi POX expression in CNP treated seedlings was 1.07, 2.03, and 2.79 folds higher than that of Chitosan treated, CNP + cPTIO treated and untreated control seedlings respectively (Fig. 9b).

PPO gene expression gradually increased following pathogen inoculation and the expression peaked at 24 hpi. In CNP treated seedlings maximum PPO expression was recorded at 24 hpi which was 1.12, 1.65, and 4.76 folds higher than that of Chitosan treated, CNP + cPTIO treated and untreated control seedlings respectively (Fig. 9c).

SOD expression gradually increased following pathogen inoculation and maximum expression was recorded at 6 hpi in treated seedlings, whereas in control seedlings maximum expression was observed at 9 hpi. SOD expression in CNP treated seedlings at 6 hpi was 1.10, 1.81, and 3.47 folds higher than that of Chitosan treated, CNP + cPTIO treated and untreated control seedlings respectively (Fig. 9d).

CAT gene expression gradually increased after pathogen inoculation and the transcript accumulation peaked at 6 hpi in treated seedlings, whereas in control seedlings maximum expression was observed at 9 hpi. CAT gene expression at 6 hpi in CNP treated seedlings was 1.11, 2.58, and 3.65 folds higher that of Chitosan treated, CNP + cPTIO treated and untreated control seedlings respectively (Fig. 9e).

**Figure 10:**
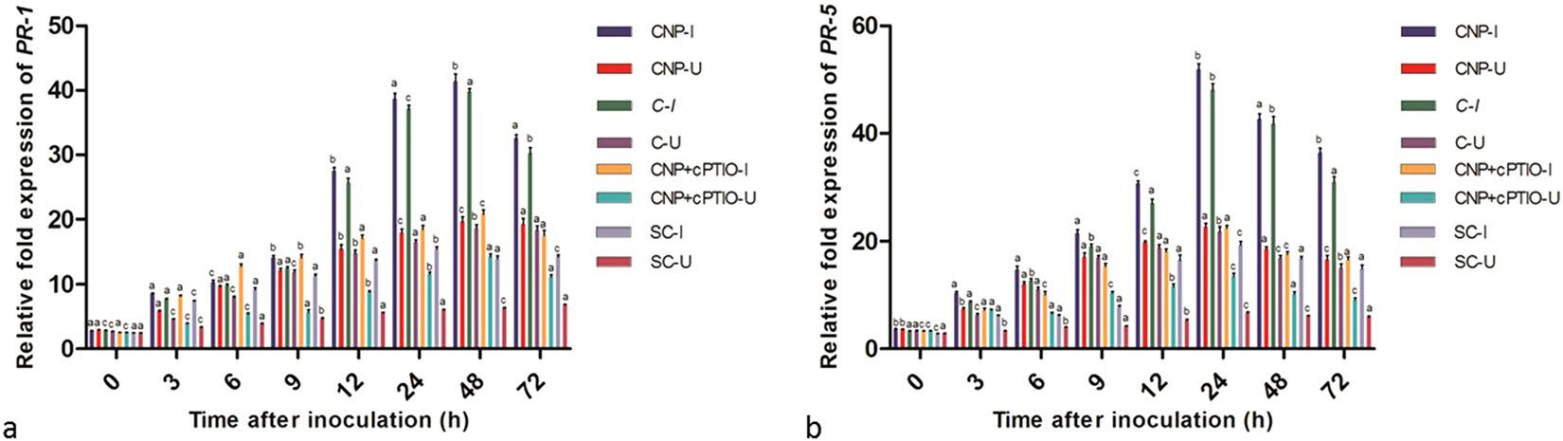
qRT-PCR determined relative expression of genes of (**a**) PR-1 and (**b**) PR-5 in two-day-old pearl millet seedlings with (I) or without (U) *Sclerospora graminicola* inoculation harvested 0, 3, 6, 9, 12, 24, 48, and 72 h. Chitosan-Seedlings treated with Chitosan, CNP-Seedlings treated with the chitosan nanoparticles, CNP + cPTIO – seedlings treated with CNP followed by cPTIO treatment, Control: Seedlings of downy mildew susceptible cultivar. Expression levels were measured by qPCR and normalized to the constitutive PP2A gene. Values are means of a single experiment carried out in triplicate. The bars indicate ± SE and the data were analyzed by one-way ANOVA followed by Tukey’s test and p-value * < or = 0.05 was significant compared with control and ** < 0.01 significant with treated control.

PR-1 gene expression gradually increased after pathogen inoculation and the expression level peaked at 48 hpi. CNP treated seedlings at 48 hpi recorded 1.04, 1.99, and 2.96 folds higher PR-1 expression than that of Chitosan treated, CNP + cPTIO treated and untreated control seedlings respectively (Fig. 10a).

PR-5 gene expression gradually increased following pathogen inoculation and maximum expression was recorded at 24 hpi. CNP treated seedlings at 24 hpi recorded 1.08, 2.31, and 2.68 folds higher PR-5 expression than that of Chitosan treated, CNP + cPTIO treated and untreated control seedlings respectively (Fig. 10b).

## Discussion

A wide array of biotic and abiotic substances is known to act as elicitors of plant defense responses imparting increased resistance against a range of plant pathogens, and this phenomenon of elicitor stimulated plant defenses is broadly termed as induced resistance^12,13^. Inducing resistance is fast gaining worldwide importance and acceptance as an eco-friendly and safe approach of plant disease management. Adoption of this plant disease control mechanism aims at reducing the risks of chemical application which are otherwise harmful to human and environmental health. With this background the present study aimed at evaluation of nanochitosan particles for their resistance eliciting efficiency against pearl millet downy mildew disease and decipher some of the important biochemical and molecular events involved in the process.

The results of the present study clearly showed that nanochitosan particles are potent elicitors of systemic resistance against pearl millet downy mildew disease. The level of disease resistance induced correlated with the enhanced enzyme activities and gene expression of defense enzymes like phenylalanine ammonia lyase, peroxidase and polyphenoloxidase. Enzymes play an important role in lowering the ROS levels and helping avoid oxidative stress. Superoxide dismutase and catalase activities and gene expression were also significantly enhanced over the control. Important markers of induced resistance like PR-1 and PR-5 were substantially overexpressed in nanochitosan particles treated pearl millet plants. The involvement of NO in nanochitosan mediated induced resistance was also demonstrated in this study.

Of the different concentration of chitosan nanoparticles evaluated in this study, it was found that 250 mg/kg seed concentration treatment for 6 hours produced the maximum seed germination and seedling vigor which was significantly higher in comparison to the chitosan treatment and untreated control. Therefore, this concentration and time of treatment was used for all further studies. Chitosan is known to quickly penetrate the seed coats and teguments and positively influences the cellular metabolism at the seed development stages^14^ resulting in enhancing the seed growth parameters like the seed germination, seedling vigor, shoot and root length and also increased biomass^15^. Similar results have been reported in maize, rice, wheat and millet crops wherein chitosan treatment stimulated the seed germination and seedling vigor significantly over the control^16–19^. Chitosan treatment increased seed germination, plant growth and yield in soybean^20^. Chitosan nanoparticles to rice seeds resulted in enhanced seed growth parameters^21^.

Chitosan nanoparticles when treated to pearl millet seeds and evaluated against downy mildew disease under greenhouse conditons provided significant downy mildew control over the untreated control. The level of protection offered by nanochitosan was on par with the Chitosan treatment though the amount of nanochitosan used was very little though there are several reports of chitosan induced resistance against plant diseases, use of nanochitosan particles in plant disease management are very few. Systemic resistance was elicited in grapevine against *Botrytis cinerea* and *Plasmopara viticola* by Chitosan and 60 and 70% infection was reduced respectively^22^. Resistance against *Verticillium* wilt of potato caused by *Verticillium dahliae* was elicited by chitosan resulting in effective control of the disease under greenhouse conditons^23^. Tea leaves treated with chitosan nanoparticles significantly enhanced disease resistance through induction of systemic resistance^6^. Early blight and Fusarium wilt of tomato were significantly reduced due to Cu-chitosan nanoparticles^7^. Blast disease of rice and finger millet were effectively suppressed by Chitosan nanoparticles^8,24^.

It is well demonstrated that the concentration of chitosan and the treatment time are very important for the achieving increased plant defense stimulation, and particularly treatment of chitosan prior to pathogen infection has been proven to be highly effective than applying chitosan after pathogen infection^25^. Further, duration of treatment varies depending upon the type of elicitor and the host-pathogen system. Varied time requirements by chitosan treatments for development of optimum defense stimulation ability was recorded in earlier studies in different plant-pathogen systems wherein tobacco-TMV interactions^26^ required one-day time interval and rape seed *Sclerotinia sclerotiorum* interactions required three-day interval^27^. In this study, chitosan nanoparticles at 250 mg kg^−1^ concentration were prior-treated to pearl millet seeds for 6 h followed by pathogen inoculation, and it was shown this treatment required a time of 2 days between treatment and pathogen inoculation for complete development of resistance which was sustained thereafter. It is important to note here that, in our previous studies we have demonstrated that chitosan seed treatment to pearl millet seeds induced systemic and durable resistance against downy mildew disease and there was a time gap of three days between chitosan treatment and downy mildew pathogen inoculation for maximum buildup of resistance. The reduced time requirement by nanochitosan to develop optimum resistance against *S. graminicola* might be attributed to the nano-nature of the chitosan particles which might have moved through the tissues rather rapidly and stimulated the necessary defense mechanisms.

Subsequent to elicitor recognition, defense signaling and transducing pathways get activated which leads to elevated production of a cascade of defense reactions like production of active oxygen species, synthesis of phytoalexins, cell wall strengthening, synthesis of defense enzymes, and the accumulation of pathogenesis-related (PR) proteins^28^. Mechanism of Chitosan induced resistance is well studied in various host-pathogen systems and it is established that chitosan treatment is known to contain the invading pathogens by forming chemical and mechanical barriers and also by enhancing the synthesis of phytoalexins, defense enzymes and PR-proteins^29,30^.

In the present study it was shown that the activities of various defense enzymes like phenylalanine ammonia lyase, peroxidase, polyphenol oxidase, superoxide dismutase and catalase were significantly enhanced in chitosan nanoparticles treated pearl millet in comparison to the control seedlings. It was also observed that the level of enzyme activities in chitosan nanoparticles treated seedlings was on par with the chitosan treatment. Further, the activities of defense enzymes were increased significantly to a higher level after the inoculation of the downy mildew pathogen. Our results are in line with previous reports which have showed increased defense enzyme activities during chitosan induced resistance in different crops. Chitosan treatment effectively controlled pitch canker caused by *Fusarium circinatum* in *Pinus patula* by stimulating innate immunity and this was associated with increased transcript accumulation of genes of defense enzyme PAL^31^. Activities of PAL, POX and PPO were significantly enhanced over the controls in sunflower and wheat plants treated with chitosan resulting in activation of systemic resistance against downy mildew of sunflower and root and foot rot of wheat^32,33^.

Activation of the pathogenesis related proteins PR-1, PR-2, and PR-5 and their pattern of expression are long been considered as importnat markers of systemic acquired resistance signaled via the SA pathway. The results of the present study clearly showed that, both PR-1 and PR-5 were activated early and in significantly higher amounts in the nanochitosan treated seedlings compared to the untreated seedlings; and the increase was more pronounced after the inoculation of the downy mildew pathogen. Further, the increase in expression of PR proteins were on par with the chitosan treatment. Similar observations of PR protein expression have been reported earlier in many crop systems. Increased PR proteins were activated during chitosan induced resistance against *Botrytis cinerea* and *Plasmopara viticola* in grapevines^22^. Chitosan oligosaccharides induced resistance to tobacco mosaic virus (TMV) in *Arabidopsis* by activating the SA singalling pathway and the resistance was associated with the increased expression of PR-1 gene^34^.

Involvement of NO in plant disease resistance elicitation has been well established in several host-pathogen systems using various elicitors^35,36^. Chitosan induced resistance against different plant pathogens has also suggested a possible role for NO in mediating the resistance elicitation. Chitosan oligosaccharide induced resistance against TMV in Arabidopsis was shown to mediated by increased NO generation acting as early signaling molecule upstream of SA-dependent defense signaling^34^. The results of the present study demonstrated that induction of resistance by chitosan nanoparticles was through NO signaling and nanochitosan treated pearl millet treated seedlings showed significantly higher generation of NO compared to the untreated control and the seedlings treated with nanochitosan along with the NO scavenger cPTIO showed significantly lesser NO. This variation in NO generation in nanochitosan treated and CNP + cPTIO treated seedlings positively correlated with the level of activities of various defense enzymes and expression of PR proteins and also the degree of downy mildew resistance achieved under field conditions. Further, in our earlier studies, chitosan induced resistance against pearl millet downy mildew was also shown to be mediated by NO^37^. Chitosan induced NO generation in *Brassica napus* leaves^38^.

## Conclusion

In conclusion, this study clearly showed that pearl millet seed treatment with nanochitosan particles will significantly promote seed germination and seedling vigor and also effectively induced systemic and durable resistance against downy mildew disease under greenhouse conditions. The induced resistance correlated with the enhanced activities and gene expression of phenylalanine ammonia lyase, peroxidase, polyphenol oxidase, superoxide dismutase and catalase. PR-1 and PR-5 gene expression were significantly higher in the nanochitosan treated seedlings compared to the untreated seedlings. The resistance elicited by nanochitosan treated was found to be mediated by NO and scavenging of NO resulted in complete reduction of disease protection. Though the amount of nanochitosan used for seed treatments was very less, its effect on the resistance elicitation and the associated biochemical and molecular mechanism was on par with the chitosan treatment which is comparatively required in higher amounts. The findings of the present study can be extended to other crop-systems wherein nanochitosan can be used as a simple, inexpensive and effective strategy for plant disease management.

## Materials and Methods

### Host

Seeds of pearl millet cultivars 7042 S and AIMP 92901-P3, highly susceptible and highly resistant to *S. graminicola*, respectively, were obtained from the International Crop Research Institute for Semi-Arid Tropics (ICRISAT), Hyderabad, India, and the All India Co-ordinated Research Project on Pearl Millet (AICRP-PM), Mandor, Jodhpur, India.

### Source of pathogen and inoculum preparation

*Sclerospora graminicola* was isolated from severely infected pearl millet cv. 7042 S grown under field conditions^39^. Leaves showing profuse sporulation of *S. graminicola* on the abaxial side were collected in the evening hours and thoroughly washed under running tap water to remove sporangia. The leaves were then blotted dry, cut into small pieces, and maintained in a moist chamber to promote sporulation. The following morning fresh sporangia were washed into distilled water. For use as inoculum, the resulting zoospore concentration was adjusted to 40,000 zoospores/ml using a hemocytometer.

### Preparation of chitosan nanoparticles (CNP)

Preparation of low molecular weight chitosan (LMWC) was done according to the procedure described by Tharanathan and Harish Prashanth^10^. Chitosan solution (~80% deacetylated chitosan,; viscosity 1% solution having viscosity average molecular weight ~110 kDa), was taken in a three-necked flat-bottomed flask, are purged with nitrogen at 60 °C with constant stirring at 200 rpm. Subsequently, potassium persulfate (KPS, 0.8 mM) was added to the solution and the reaction was completed in 2 h. The reaction mixture was precipitated with isopropyl alcohol (3 volumes) to get LMWC with MW of ~20 kDa, ~70 acetylated (characterized by HPLC and ^13^CNMR respectively, Fig. 1), re-dissolved in deionized water, dialyzed (dialysis membrane -150, Sigma Chemical Co., MO, USA) overnight and lyophilized (Virtis, Gardiner, NY, USA)^40^. The LMWC nanoparticles were prepared by the method of Calvo *et al*.^41^ with slight modification. Briefly LMWC (20 mg) was taken in 4 mL distilled water and 1 mL of sodium tripolyphosphate (TPP, 0.1%) solution was added slowly dropwise and stirred at 500 rpm at room temperature (27 ± 1 °C). Initially, the contents appear opalescent. To this, 100 μL of distilled water was added and stirred for 1 min to obtain clear solution of LMWC nanoparticles^11^.

### Seed treatment with chitosan nanoparticles (CNP)

CNP was treated to pearl millet as seed treatment. For seed treatment, 7042 S seeds were surface-sterilized with 0.02% mercuric chloride for 5 min, and rinsed thoroughly in SDW. LMWC solutions were prepared by dissolving LMWC in distilled water and sodium tripolyphosphate solution so as to obtain final concentrations of 50, 100, 250, and 500 mg LMWC per 100 ml, and this solution was treated at the rate of 100 ml kg^−1^ seed. Seeds were coated with 1% gum arabic as an adhesive and suspended in different concentration of nanochitosan solution and kept at 25 ± 2 °C in a rotary shaker for 6 h to ensure uniform coating. Seeds of pearl millet cultivars AIMP 92901-P3 treated with distilled water and 7042 S treated with chitosan (@ 3 g kg^−1^ seed) for 3 h served as resistant and induced resistant checks respectively. 7042 S seeds treated with distilled water for the same duration served as control.

### Effect of CNP on seed germination and seedling vigor of pearl millet under laboratory conditions

CNP treated seeds and controls were seeded onto distilled water soaked brown germination paper. Fifty seeds of pearl millet were placed equidistantly on the paper. Another presoaked paper towel was placed on the first one so that the seeds were held in position. The towels were then rolled and wrapped with polythene to prevent drying. After incubation for 7 days, the towels were unrolled and the numbers of seeds germinated were counted. Seedling vigor was analyzed at the end of 7 days of incubation by the method of Abdul-Baki and Anderson^42^. The length of the root and shoot of individual seedlings was measured to determine the vigor index. The vigor index was calculated using the formula: Vigor index = (mean root length + mean shoot length) × (% germination). The experiment was carried out with four replicates of 100 seeds each and was repeated three times.

### Efficiency of CNP to elicit resistance against downy mildew under greenhouse

In the greenhouse, CNP was applied as seed treatment. 7042 S seeds treated with SDW and chitosan served as the control and induced resistance check respectively. 7042 S seeds treated with the systemic fungicide, metalaxyl (Apron 35 SD at 6 g kg^−1^ seeds) served as fungicide treated control.

The treated seeds were sown in earthen pots filled with autoclaved soil, sand and manure at the ratio of 2:1:1. Each treatment consisted of 4 replications, ten pots per replication, and ten seedlings per pot. Treatments were arranged in a randomized complete block design. Three-day-old seedlings were challenge-inoculated by the whorl inoculation method with a zoospore suspension of *S. graminicola* at a concentration of 40,000 zoospores/ml prepared as described previously^43^. In the whorl inoculation method, droplets of *S. graminicola* zoospores were dropped onto the leaf whorl formed by the emerging seedlings and allowed to flow down to the base. These pathogen-inoculated plants were maintained under greenhouse conditions (90–95% RH, 20–25 °C temperature), and observed for disease development. The plants were rated for disease when they showed any one of the typical downy mildew symptoms such as sporulation on the abaxial leaf surface, chlorosis, stunted growth, or malformation of the earheads. Downy mildew disease incidence was recorded at 30 DAS (days after sowing) and final counts were made at 60 DAS. The experiment consisted of 4 replicates of 100 seedlings each and was repeated twice.

### Demonstration of the nature of resistance induction by CNP

This included two sets of experiments. In the first set, 7042 S seeds treated with *CNP* as described above were sown in earthen pots filled with autoclaved soil, sand and manure in the ratio 2:1:1. The emerging seedlings were challenge-inoculated with the zoospore suspension of *S. graminicola* by adding 4–5 drops (0.5 ml) to the leaf whorl of each plant at intervals of 1, 2, 3, 4, 5 and 6 days between the seedling emergence and pathogen inoculation in different sets of plants. In the second set 7042 S seeds were plated on moist blotters and were incubated at 25 + 2 °C in an incubator. Thirty-six hours later the roots of the seedlings were treated with nanochitosan by soaking the roots in the fungal spore suspension of 10^8^ cfu ml^−1^ concentration for three hours and later the seedlings were transplanted into earthen pots filled with soil, sand and manure in the ratio 2:1:1. The seedlings were then challenge-inoculated with zoospore suspension of *S. graminicola* (40,000 zoospores/ml) following the whorl inoculation procedure with a time gap of 1, 2, 3, 4, 5 and 6 days in different sets of plants. 7042 S seeds treated with distilled water was maintained as control for both the above sets of experiments^44^. The experiment consisted of 4 replicates of 100 seedlings each and was repeated twice. All the above sets of plants were maintained under greenhouse conditions, observed for the downy mildew disease reaction, and downy mildew disease data recorded as described earlier and the disease protection (%) was calculated as follows:

$${\rm{Downy}}\,{\rm{mildew}}\,{\rm{disease}}\,{\rm{protection}}=\frac{{\rm{C}}-{\rm{T}}}{{\rm{C}}}\times 100$$

where, C, is percent downy mildew disease incidence in control; T - percent downy mildew disease incidence in treated plants (Safeeulla)^39^.

### Biochemical and gene expression studies

#### Plating of treated seeds

7042 S seed treatments with CNP and chitosan were same as described above for germination studies. In addition, AIMP 92901-P3 seeds treated with distilled water served as resistant check. After treatment, the seeds were plated on pre-soaked blotters in perspex plates and incubated for two days.

#### Challenge inoculation and harvesting of seedlings

Two-day-old seedlings were root-dip inoculated with a zoospore suspension of 40,000 zoospores/ml, and incubated in dark at 25 ± 2 °C. One set of the treated seedlings were inoculated with sterile distilled water which served as uninoculated control. A total of 1 g seedlings for each experiment in three replicates were harvested at 0, 3, 6, 9, 12, 24, 48 and 72 h after inoculation (hai) and immediately wrapped in aluminum foil and stored at −80 °C until further use for enzyme assays and RT-PCR analysis.

## Biochemical studies

### Enzyme assays

#### Enzyme extraction

Harvested pearl millet seedlings (1 g fresh weight) were ground to a fine paste in 1 ml of buffer. The extract was centrifuged at 12,000 g for 20 min at 4 °C and the supernatant was transferred to a new tube and used as the enzyme extract.

#### Protein estimation – Lowry’s method

To calculate the specific activity of the enzymes, protein content in the crude extract was estimated by Lowry’s method using BSA (Sigma) as a standard^45^.

#### Phenylalanine ammonia-lyase assay

PAL enzyme was extracted with 25 mM Tris HCl buffer (pH 8.8). PAL activity was assayed according to the procedure of Beaudoin-Eagan and Thorpe^46^. One hundred microlitres of extracts were mixed with 900 ml of 50 mM L-Phenylalanine and 100 mM Tris HCl buffer solution (pH 8.01). The mixture was placed in a water bath at 40 °C for 120 min. The reaction was stopped by adding 60 ml of 5 N HCl. Enzyme activity was determined as the amount of t-cinnamic acid formed from L-Phenylalanine per mg of protein per min measured spectrophotometrically at a wavelength of 290 nm.

#### Peroxidase assay

POX enzyme was extracted in 10 mM potassium phosphate buffer (pH 6.9). POX activity was assayed according to the procedure of Hammerschmidt *et al*.^47^. The reaction mixture (3 ml) consisted of 0.25% v/v guaiacol in 10 mM potassium phosphate buffer (pH 6.0) containing 100 mM hydrogen peroxidase. The crude enzyme (10 mL) was added to initiate the reaction, which was measured spectrophotometrically at 470 nm. One unit of POX enzyme activity is defined as the increase in absorbance recorded 470 nm. POX activity is expressed in terms of the change in A_470_ min^−1^ mg^−1^ protein.

#### Polyphenol oxidase assay

PPO enzyme was extracted in Tris–HCL buffer (pH 7.0) containing 0.1 M KCl, 1% (v/v) TritonX-100,1 mM EDTA and 5% (w/v) Polyvinylpolypyrrolidone (PVPP). The reaction mixture (3 ml) consisted of 10 mM catechol in 100 mM potassium phosphate buffer (pH 6.5) was assayed as described by Arora and Bajaj^48^. The standard reaction mixture consisted of 3 ml of 10 mM sublimated catechol in 100 mM potassium phosphate buffer (pH 6.5) and 10 ml of enzyme extract. Increase in absorbance at 420 nm was recorded for 1 min. The results are expressed as the change in A per min per mg protein.

#### Superoxide dismutase assay

SOD enzyme was extracted in 50 mm potassium phosphate buffer (pH 7·0). The activity of SOD was assayed by measuring its ability to inhibit the photochemical reduction of nitroblue tetrazolium (NBT) using the method of Beauchamp and Fridovich^49^. The 3 mL reaction mixture contained 50 mm phosphate buffer, pH 7·8, 13 mm methionine, 75 µm NBT, 2 µm riboflavin, 0·1 mm EDTA, and 150 µL enzyme extract. Riboflavin was added at the end and the tubes were shaken and placed 30 cm below a light source consisting of two 15 W fluorescent lamps. The reaction was started by switching on the light and was allowed to run for 15 min. The reaction was stopped by switching off the light and the tubes were covered with a black cloth. The absorbance of the reaction mixture was read at 560 nm. A nonirradiated reaction mixture did not develop colour and served as control.

#### Catalase assay

Catalase was extracted in sodium phosphate buffer (68 mM, pH 6.8) and assayed following the method of Bailly *et al*.^50^. The reaction mixture contained 3 ml of phosphate buffer along with 40 ml crude enzyme extract. The reaction was initiated by adding 40 ml of 10 mM H_2_O_2_ and which was measured spectrophotometrically at 240 nm (Hitachi U 2000, Japan). Catalase activity was expressed in terms of the change in absorbance at 240 nm in the linear phase of the slope (D240 min^1^ mg^1^ protein).

## Gene expression studies

### Quantitative real-time PCR analysis (qPCR) for defense enzymes, and pathogenesis-related proteins

#### RNA extraction

A total of 100 mg of frozen seedlings was ground to fine powder in a 2 ml SealRite microcentrifuge tube using stainless steel beads and an automated shaker SO-10 M (Fluid Management, Wheeling, IL, USA). Total RNA was extracted from seedlings harvested at different times noted above by using the RNeasy plant mini Kit (Qiagen) as per the manufacturer’s instructions. Eluted RNA was stored at −80 °C and then treated with DNase I (RNase free) (Fermentas). The concentration and purity of RNA was determined by means of spectrophotometer and its integrity by agarose gel electrophoresis.

#### RT-PCR analysis

The relative quantitation of PAL (NM001174615.1), POX (EU492461), PPO (AY881993.1), SOD (AY823553.1), CAT (JN627402.1), PR1 (HQ699781.1), and PR5 (EU725133.1) *mRNAs* in pearl millet seedlings was done by using gene-specific primers^51^, designed with Primer Express version 3.0 software (Applied Biosystems) (Table 1). PP2A (protein phosphatase 2A) served as endogenous reference gene. Primer specificities were confirmed by agarose gel electrophoresis of the RT-PCR products. Each qPCR reaction (20 μL) consisted of 1 × SYBR Green PCR master mix (SYBR Green mix, Applied Biosystems), 3 pmol of each primer and 20 ng each of cDNA and used StepOnePlus™ Real-Time PCR Systems (Applied Biosystems). qPCR steps were: denaturation at 95 °C for 10 min, 40 cycles of 15 s at 95 °C, 60 s at 60 °C. At the end of each reaction, a melting curve was created using a single cycle consisting of 15 s at 95 °C and 60 s at 60 °C. This was followed by a slow temperature increase to 95 °C at the rate of 0.3 °C s^−1^. The quantification of target mRNAs used a comparative Ct method^52^.

**Table 1:** Primer sequences used for qRT-PCR amplification.

Sl. No.	Target gene amplified	Forward primer sequence (5′ to 3′)	Reverse primer sequence (5′ to 3′)
1	Phenyl alanine ammonia lyase	ATGGAGTGCGAGAACGGCC	CTGCGCGATGCTGAGGCT
2	Peroxidase	CCCCAGAAGCACATTTGTGA	CATGGCTGCGGGCGGAG
3	Polyphenol oxidase	AGTCGAGGTTTGGCCACCAT	CCACCTGATGCGCTCGATG
4	Superoxide dismutase		
5	Catalase	GGCAAGTCCCACTACGTCAA	AGCTGCTCGTTCTCGTTGAA
6	PR1	TGGACGTGCCGCTGCCG	GAACTGCGCCGCCACACG
7	PR-5	GCGTCCTCGGTCCTCCTG	CACACGCGGCCGGAGCTG
Reference housekeeping gene			
8	Protein phosphatase 2 A	TGAGAGCAGACAAATCACTCAA	AAGAGCTGTGAGAGGCAAATAA

**Table 2:** Effect of pearl millet seed treatment with different concentrations of CNP on seed germination and seedling vigour under laboratory conditions.

Treatment	Concentration	Germination (%)	Seedling vigor
CNP	50 mg kg^(−1)^	94 ± 2.1^b^	1918 ± 4.4^b^
	100 mg kg^(−1)^	95 ± 1.3^ab^	1922 ± 3.3^b^
	250 mg kg^(−1)^	97 ± 1.8^a^	1937 ± 4.1^a^
	500 mg kg^(−1)^	93 ± 1.3^b^	1905 ± 4.6^c^
Chitosan	3 g kg^(−1)^	89 ± 1.9^c^	1917 ± 4.1^c^
Control	Untreated	82 ± 1.8^c^	1866 ± 3.9^cd^

Percentages of seed germination and vigour index are mean from three repeated experiments. Vigour index was calculated on percentage germination and mean root and shoot lengths of the seedlings. The values are mean from three experiments. Means designated with the same letter in column are not significantly different according to Tukey’s HSD test at P = 0.05.

### Data analysis

Germination experiments were carried out with four replicates and were repeated thrice, greenhouse experiments were carried out in four replicates and were repeated twice, biochemical and molecular were carried out in three replicates and were repeated thrice. Data were analyzed separately for each experiment and were subjected to arcsine transformation and analysis of variance (JMP Software; SAS Institute Inc., Cary, NC). Significance effects of treatments were determined by the magnitude of the F value (P = 0.05). Treatment means were separated by Tukey’s honest significant difference test.
